# Sevoflurane inhibited reproductive function in male mice by reducing oxidative phosphorylation through inducing iron deficiency

**DOI:** 10.3389/fcell.2023.1184632

**Published:** 2023-06-06

**Authors:** Xue Zhang, Yong Zuo, Jianhua Zhang, Di Zhang, Muhammad Naeem, Yanzhong Chang, Zhenhua Shi

**Affiliations:** Laboratory of Molecular Iron Metabolism, College of Life Science, Hebei Normal University, Shijiazhuang, Hebei, China

**Keywords:** sevoflurane, iron metabolism, oxidative phosphorylation, spermatogenesis, epididymis, testis

## Abstract

Sevoflurane (Sev) is one of the commonly used inhalation anesthetic chemicals in clinics. It has great impact on spermatogenesis and fertilization in male animals. The underlying mechanism remains largely unexplored. Based on our previous research, we hypothesized that Sev induced iron metabolism disturbance in the testis and epididymis and inhibited the spermatogenesis. In this study, two-month-old C57BL/6 male mice were treated with 3% Sev for 6 h, and their fertility (including sperm concentration, sperm mobility, and the number of offspring) was evaluated. Mice testis, epididymis, and sperm were harvested and subjected to Western blot analysis and immunofluorescence analysis. Iron levels were reflected by the gene expression of iron metabolism-related proteins (including ferritin, TfR1, and FpN1) and ICP-MS and Perl’s iron staining. Electron transport and oxidative phosphorylation levels were measured by Oxygraph-2k and ATP contents. The activity of ribonucleotide reductase was evaluated by assay kit. DNA synthesis status in testis and/or epididymis was marked with BrdU. Cell proliferation was evaluated by double immunofluorescence staining of specific protein marker expression. Our results revealed that the mice exposed to Sev showed damaged testicular and epididymis structure and significantly reduced the sperm concentration, sperm motility, and fertility. Sev decreases the iron levels through down-regulating the expression of H-ferritin, L-ferritin, and FpN1, and up-regulating the expression of TfR1 in the testis and epididymis. Iron levels also significantly reduced in germ cells which decrease the number of germ cells, including sperm, Sertoli cells, and primary spermatocyte. Iron deficiency not only decreases electron transport, oxidative phosphorylation level, and ATP production but also suppresses the activity of ribonucleotide reductase and the expression of Ki67, DDX4, GATA1, and SCP3, indicating that Sev affects the spermatogenesis and development. Meanwhile, Sev impaired the blood-testis barrier by decreasing the ZO1 expression in the testis and epididymis. The damage effect induced by Sev can be significantly ameliorated by iron supplementation. In conclusion, our study illustrates a new mechanism by which Sev inhibits spermatogenesis and fertility through an oxidative phosphorylation pathway due to iron deficiency of epididymis and testis or sperm. Furthermore, the damaging effects could be ameliorated by iron supplementation.

## Introduction

The quality and quantity of sperm in male mammals, including humans, are key indicators of fertility. Spermatogenesis of healthy sperm determines the quantity and quality of sperm. Spermatogenesis and mature spermatozoa development are very complex and tightly regulated processes. The differentiation of spermatogonia into spermatozoa requires the participation of several cell types, hormones, paracrine factors, genes, and epigenetic regulators (Neto et al., 2016). Therefore, an imbalance in the regulation of any link will affect the quantity and quality of sperm and ultimately affect fertility.

Exposure to environmental chemicals affects the spermatogenesis. Sperm formation requires the meiosis, which is sensitive to environmental factors such as radiation and chemicals (Kesari et al., 2018) (Bhardwaj et al., 2021). Sevoflurane (Sev) is one of the commonly used inhalation anesthetic chemicals in clinics. Although its safety has been widely recognized, some of the side effects of its use have received constant attention in recent years. Sev acting on the central nervous system induces cognitive impairment (Belrose and Noppens, 2019; Chen et al., 2020). In the peripheral system, Sev induces disorders of spermatogenesis by causing the imbalance of sex hormones in the hypothalamic-pituitary gonadal axis (Cui et al., 2019). Interestingly, exposure to Sev can induce the spatial memory impairment in the next-generation of neonatal male mice through epigenetic modification (Ju et al., 2018), which suggests that Sev may also affect sperm formation and development, which further influence neurodevelopmental abnormalities in offspring.

Iron is an important cofactor of enzymes involved in many biochemical reactions. Since iron is a key component of iron–sulfur cluster and cytochrome, iron deficiency can affect ATP synthesis. Most ATP in cells is produced during electron transport in the respiratory chain. ATP is the energy source for sperm motility, one of the key indicators of sperm quality. Hence, iron metabolism dysfunction can affect spermatogenesis and development (Tsao et al., 2022). We have found that Sev can cause cognitive deficits in elderly mice or offspring of pregnant mice by inducing disturbances in brain iron metabolism (Zuo et al., 2020; Ge et al., 2021; Wang et al., 2021). In this study, we hypothesized that Sev may inhibit sperm formation and development by affecting iron metabolism disorders in testicular or epididymis tissues. Such studies would reveal the mechanism by which sevoflurane inhibits reproductive function.

## Materials and methods

### Animals and anesthetic treatment

All animal experiments were performed under ethical standards, and the Hebei Normal University ethics committee approved the procedures. Two-month-old male mice were housed under controlled illumination (12-h light/dark, lights on at 7:00 a.m.) and temperature (23–24°C) with free access to food and water. The mice were categorized into four groups: control group, Sev treatment group, the iron supplementation group, and the Sev + iron treatment group. According to our previous report, anesthesia and iron supplement treatment (Zuo et al., 2020). Briefly, the mice were placed in an anesthetic induction chamber filled with Sev (3%) for approximately 5 min until they became unconscious. The mice were then removed the anesthetizing apparatus with a 37°C thermostatic pad. The mice were treated with gases of 3% Sev and 40% oxygen within the anesthetic chamber for 6 h and were continuously monitored (Ohmeda Excel 210 SE anesthetic machine, Datex Instrumentarium Corp, Helsinki, Finland). Mice were killed after 12 h of recovery from the effects of the anesthetic for subsequent experiments. For the iron supplementation group, 1-month old mice had free access to water containing 10 mg/L of ferric chloride for 30 days. Then, the mice were anesthetized according to the method above.

### Assay of fertility, sperm number, and sperm mobility

Male fertility assay was performed by comparing the number of offspring produced by females. The anesthetized male mice were immediately paired with female mice to give birth to their first litter. After 15 days of sevoflurane anesthesia, both male and female mice were housed together and gave birth to the second litter. For the assay of sperm number, the tail of epididymis of mice was removed, and the tail semen was extruded with two pointed tweezers and placed in 1 mL normal saline. The suspension was placed in an oven at 37°C for 10 min. Ten microliters of the above suspension were drawn to the cell counting plate, the number of sperm in 5 squares was counted, denoted as R, and then the number of sperm in each epididymal sperm suspension according to the formula Rx50000 = sperm number (million/mL) was calculated. During the sperm suspension count, sperm motility count was carried out at the same time, and the number of motile sperm in 200 sperm was counted under high magnification.

### H and E staining

H and E staining was prepared according to protocols followed by Abidalla (Abidalla and Battaglia, 2019). All staining was carried out at room temperature (24°C) and slices of testis and epididymis were stained in Harris’ hematoxylin for 6 min. The samples were washed with distilled water for 5 min and then were differentiated in acid ethanol for 1–3 s. The samples were washed with distilled water for 5 min and stained with 1% (w/v) eosin for 2 min. Then, the slices were washed with running tap water, dehydrated through the alcohols, and cleared in xylene. Finally, the slices were mounted in Canadian balsam.

### Perl's iron staining of testis and epididymis

The slices of testis and epididymis were taken out at −80°C from freezer and then returned to room temperature. The slices were washed with 0.01 mol/L PBS (pH 7.4) for three times, for 5 min each, and then the slices were incubated with 3% H_2_O_2_ for 20 min. The slices were washed with 0.01 mol/L PBS (pH7.4) for three times, 5 min each again. The slices were stained using Perl’s dye liquor for 8 h and then were washed with 0.01 mol/L PBS (pH7.4) for three times, 5 min each. Finally, the slices were strengthened with dyeing with DAB-H2O2 solution and then were washed with double-distilled water for 30 min. After being washed with the gradient of ethanol and xylene, the slices were packaged with neutral gum, and the images were taken with the microscope (ZEISS Axio imager).

### Immunofluorescence assay

The mice were anesthetized using the 0.4% pentobarbital sodium (1 mL/100 g) solution. After that, the mice were perfused with 0.9% saline solution and treated with 4% paraformaldehyde. The testis and epididymis were carefully dissected and immersed in 30% sucrose solution for 2 days. The testis and epididymis were sliced into 15-μm-thick slices, and then the slices were washed with 0.01 M PBS three times for 5 min each time. The slices were then incubated with goat serum at 37°C for 60 min. Mouse monoclonal FtH antibody (1:200; Cat. No. ab104224, Abcam, Waltham, MA, United States), anti-FtL (1:400; Cat. No. 20403-1-AP ProteinTech, Rosemont, IL, United States), anti-DDX4 (1:400; Cat. No. CL488-67147, ProteinTech, Wuhan, China), anti-SCP3(1:400; No. ab97672, Abcam, Waltham, MA, United States), anti-GATA1 (1:400; Cat. No. 60011-1-Ig, ProteinTech, Wuhan, China), anti-Ki67 (1:400; No. ab115730 Abcam, Waltham, MA, United States), and anti-ZO1 (1:400; Cat. No. ab24950, Abcam, Waltham, MA, United States) were used as primary antibodies. The slices were incubated overnight at 4°C. Next, the slices were incubated with FITC-conjugated and rhodamine-conjugated secondary antibodies. Images were observed using a ZEISS LSM710.

### Assay of flux of oxygen, electron transport (ET) capacity and oxidative phosphorylation (OXPHOS) capacity

Fresh testis tissues (10 mg) were immersed in 2 mL ice-cold mitochondrial respiration buffer, containing 110 mM sucrose, 60 mM K-lactobionate, 0.5 mM EGTA, 1 g/L BSA essentially fatty acid free, 3 mM MgCl2, 20 mM taurine, 10 mM KH2PO4 and 20 mM HEPES adjusted to pH 7.1 at 37°C. Using a pair of sharp forceps, tissues were cut into small pieces and transferred with the medium into an FT500-PS Shredder Pulse Tube for use with the PBI- shredder (Oroboros Instruments, Innsbruck, Austria). Tissue-dependent homogenization was then carried out according to the manufacturer’s instructions. The Oxygraph-2k (O2k, OROBOROS Instruments, Innsbruck, Austria) was used for measurements of flux of oxygen, electron transport (ET) capacity and OXPHOS capacity in 2-mL chambers (Abdel-Rahman et al., 2016). Before starting the experiment, calibration at air saturation versus zero oxygen was performed by allowing the respiration medium (MIR05 buffer), the buffer to equilibrate with air in the oxygraph chambers and stirred at 540–560 rpm for 30–40 min, until a stable signal was detected. 2 mL of testes homogenate was then added to each chamber, and tissues were permeabilized by the addition of saponin (50 μg/mL). The mitochondrial (mt) and substrate–uncoupler–inhibitor titration (SUIT) protocol were performed as follows: Mitochondrial→ Pyruvate and Malate (PM) →ADP (D) →Oligomycin (Omy) →Carbonyl cyanide 4-(trifluoromethoxy)phenylhydrazone (FCCP, U)→Rotenone→ Antimycin A (Ama). The rates of oxygen consumption were calculated as the negative time derivative of oxygen concentration. Electron transport and OXPHOS capacity through complex I and III were abolished by adding rotenone (0.5 mM) and antimycin A (2.5 mM), respectively. Data acquisition and analysis were performed with the DatLab R software, version 4.3 (Oroboros Instruments).

### Measurement of ATP contents

ATP content of epididymis was tested by using the ATP assay kit (S0026, Beyotime, China). Briefly, epididymis tissues were lysed by ATP-releasing reagent. Then the extracts were mixed with ATP detection solution which contained luciferase, and Synergy HT luminescence plate reader detected the bioluminescence. ATP content was evaluated according to the standard curve. Results were normalized to tissue protein concentration and determined by an enhanced BCA Protein Assay kit (Beyotime, China).

### Ribonucleotide reductase (RNR) activity assay

According to the instruction, the RNR activity test of testis was carried out using kit from Mlbio company (NO. YJ151420, Shanghai, China).

### Testis iron levels were measured by ICP-MS

ICP-MS assay was carried out according to the method described by Zhang et al. (2019). Briefly, control (Con) and Sev group samples were thermally digested in 70% nitric acid using ramp-to-temperature microwave method. Digested samples were diluted for mass spectrometric evaluation, and the total iron content of the samples was determined using an Agilent 7500ce ICP-MS (Agilent Technologies, Santa Clara, CA). An eight-point calibration curve was performed prior to sample analysis. At least three samples were analyzed by ICP-MS.

### Western blot analysis

The testis and epididymis tissues were homogenized in RIPA buffer followed by centrifugation at 12,000 g at 4°C for 20 min. The supernatant containing proteins was collected and measured its content using protein quantification kit (KangWei, Beijing, China). The samples were resolved by 8%–12% of SDS-PAGE, respectively, and then transferred to nitrocellulose membranes (Millipore, Bedford, MA, United States). The target proteins FtH (from Abcam, United States), FtL (from Abcam, United States), FpN1 (Alpha Diagnostic International, United States), TfR1 (Alpha Diagnostic International, United States), and ZO-1 (ThermoFisher Scientific, United States) were detected by their primary antibodies. The relative expression quantity of proteins was normalized to that of GAPDH (mouse monoclonal; Kang Wei, Beijing, China).

### Statistical analysis

Statistical analyses were conducted on raw data by using the GraphPad Software’s Prism 7 (GraphPad Software, United States) and values were reported as mean ± SD. The primary outcomes were the short-term damage to the reproductive capacity of male mice exposed to Sev. All other outcome measures were further elucidation by the mechanism. All Western blotting data were obtained based on gray values using ImageJ Software. Student’s t-test was used to analyze sperm motility capacity, sperm concentration, iron concentration, Ki67+ cell number, BrdU + cell number, ATP content, ET capacity, OXPHOS capacity, ZO-1expression and Bcl2/Bax ratio. One-way ANOVA was used to analyze sperm concentration and sperm motility of male mice exposed to sevoflurane after iron supplementation. Two-way ANOVA was used to analyze the pregnancy rate, the number of offspring, FtH expression, FtL expression, TfR1 expression and FpN1expression. A probability level of 95% (*p* < 0.05) was considered statistically significant, and significance testing was two-tailed. Finally, it is important to note that adjusted *p*-values of Bonferroni correction were calculated by dividing the accurate *p*-values by experimental size, and adjusted *p*-values were reported.

## Results

### Sev reduced short-term fertility in mice

**FIGURE 1:**
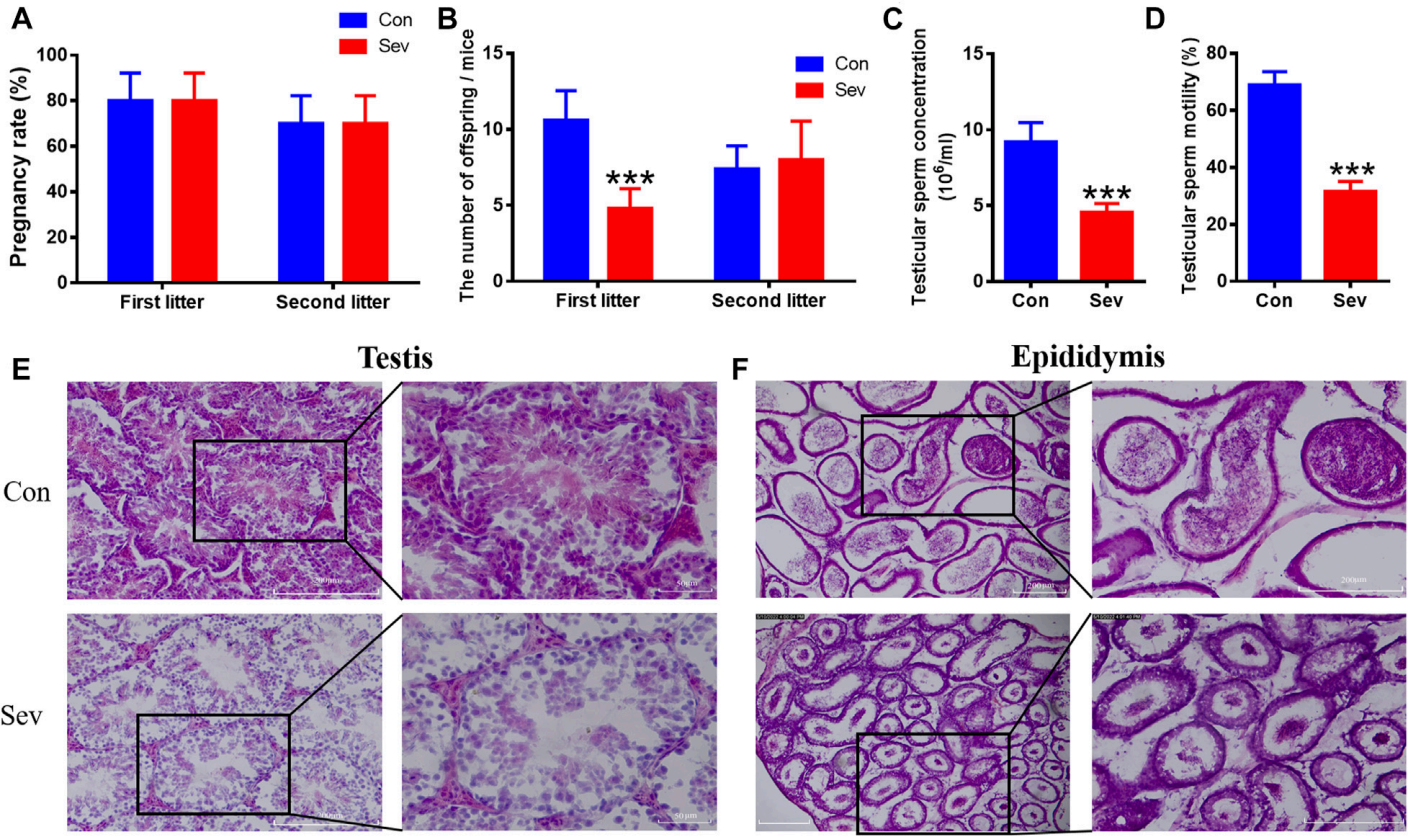
Effects of Sev on fertility, sperm concentration, sperm motility, and testicular and epididymal tissue structure in male mice. **(A)** The rate at which male mice impregnate female mice (n = 5). **(B)** The number of offspring per female mouse (n = 5). **(C)** The concentration of sperm in male mice (n = 5). **(D)** Sperm motility in the epididymis (n = 5). **(E, F)** showed the effect of Sev on testicular and epididymal tissue structure by H and E staining (n = 3, 10X: scale bar = 200 μm, 20X:scale bar = 50 μm). Data were expressed as mean ± SD. ***p < 0.001 compare to that of control, respectively.

We measured the number of offspring of Sev-treated male mice to evaluate the fertility of Sev on male mice. At the same conception rate in female mice (Figure 1A), the number of offspring of each anesthetized male mouse in the first litter was significantly reduced (Figure 1B). There was no difference in second litter compared to the control group (Figure 1B). We found that the decrease in fertility was due to Sev-induced decreased sperm count (*p* < 0.001) and decreased sperm motility (*p* < 0.001) significantly, as shown in Figures 1C,D. H and E staining results showed that Sev resulted in the vacuolization of the testis and epididymis (Figures 1E,F), indicated that Sev impaired the tissue structure of testis and epididymis.

### Sev caused iron deficiency in testis and epididymis

**FIGURE 2:**
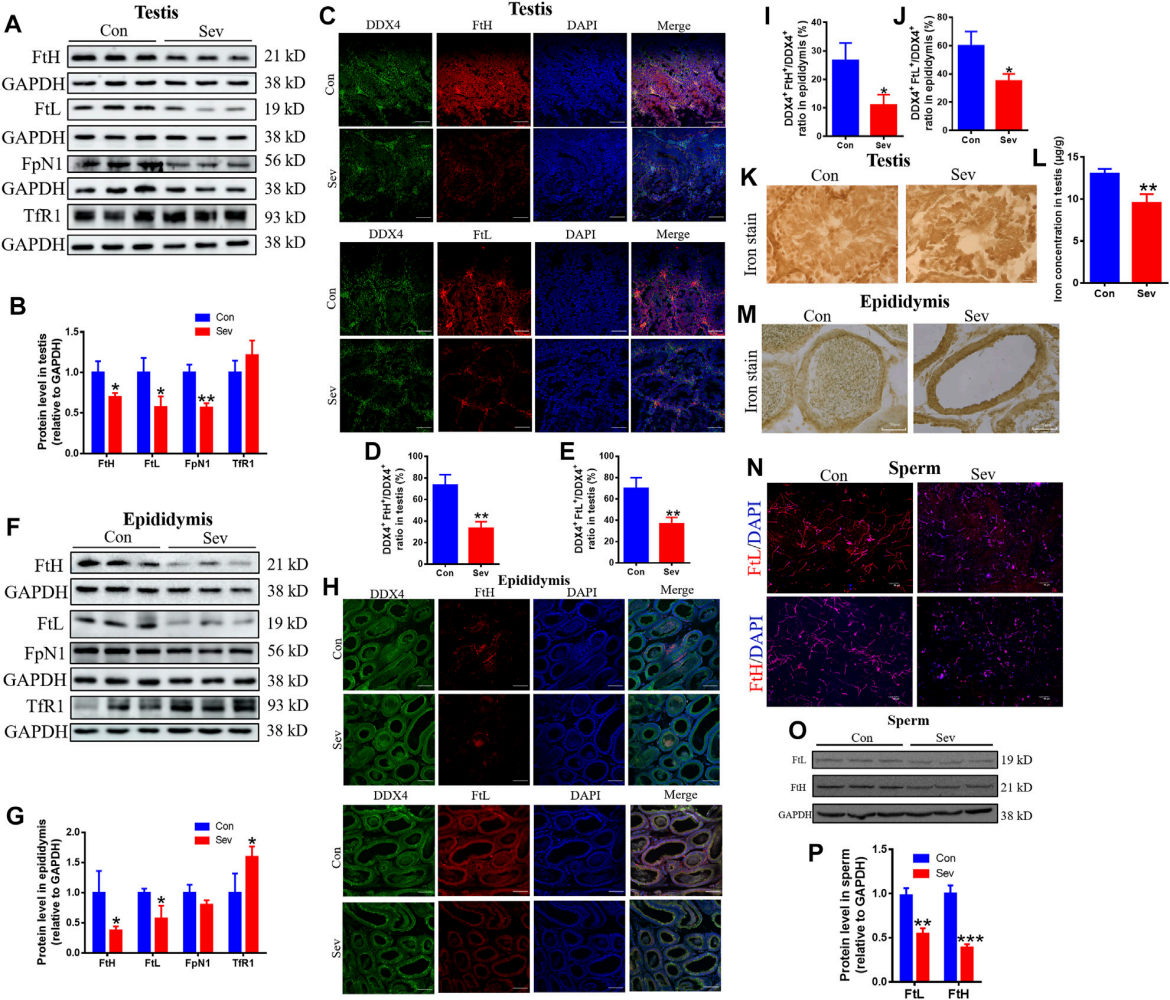
Effects of Sev on expression of iron metabolize-related proteins and iron levels in testis and epididymis **(A,B,F,G)**: The expression of FtH, FtL, FpN1 and TfR1 and statistic analysis in testis and epididymis (n = 5). **(C,H)** showed the expression of FtH and FtL in germ cells of testis and epididymis (n = 3, Scale bar = 100 μm). **(D,E,I,J)** were statistical graph of **(C,H)**. **(K,L,M)**: Iron levels in the testis and/or epididymis were measured by Perl’s staining (scale bar = 100 μm) and ICP-MS (n = 5), respectively. **(N,O,P)**: The expression of FtH and FtL in sperm detected by immunofluorescence (scale bar = 100 μm) and Western blot. Data are expressed as mean ± SD. *p < 0.05, **p < 0.01 and ***p < 0.001 compare to that of control, respectively.

The expression of ferritin, including heavy chain ferritin (FtH) and light chain ferritin (FtL), transferrin receptor1 (TfR1) and Ferroportin1(FpN1) (the only known iron output protein) reflected the iron levels of tissues (Le and Richardson, 2002; Kernan and Carcillo, 2017; Bao et al., 2021). We performed Western blot to evaluate the expression of ferritin, TfR1, and FpN1 in testis and epididymis. We found that Sev dramatically decreased the expression of FtH and FtL in testis and epididymis. FpN1 expression downregulated only in testis. TfR1 expression upregulated only in epididymis as shown in Figures 2A,B,F,G, indicating that Sev caused the iron deficiency in testis and epididymis. DDX4 was a marker of germ cells in testis and epididymis (Shiraishi et al., 2020). We further found that ferritin downregulated significantly in germ cells in testis and epididymis as shown in Figures 2C,D,E,H,I,J). In order to verify the iron levels induced by Sev, we further tested the iron concentration in testis and/or epididymis using ICP-MS and Perl’s iron staining as shown in Figures 2K,L,M). The results were consistent with that of Western blot and immunofluorescence. Iron deficiency of testis and epididymis or germ cells further caused FtH and FtL expression decrease in sperms as shown in Figures 2N,O,P, indicating that Sev caused the iron deficiency in sperms.

### The Sev-induced iron deficiency inhibited cell proliferation and promoted cellular apoptosis in testis

**FIGURE 3:**
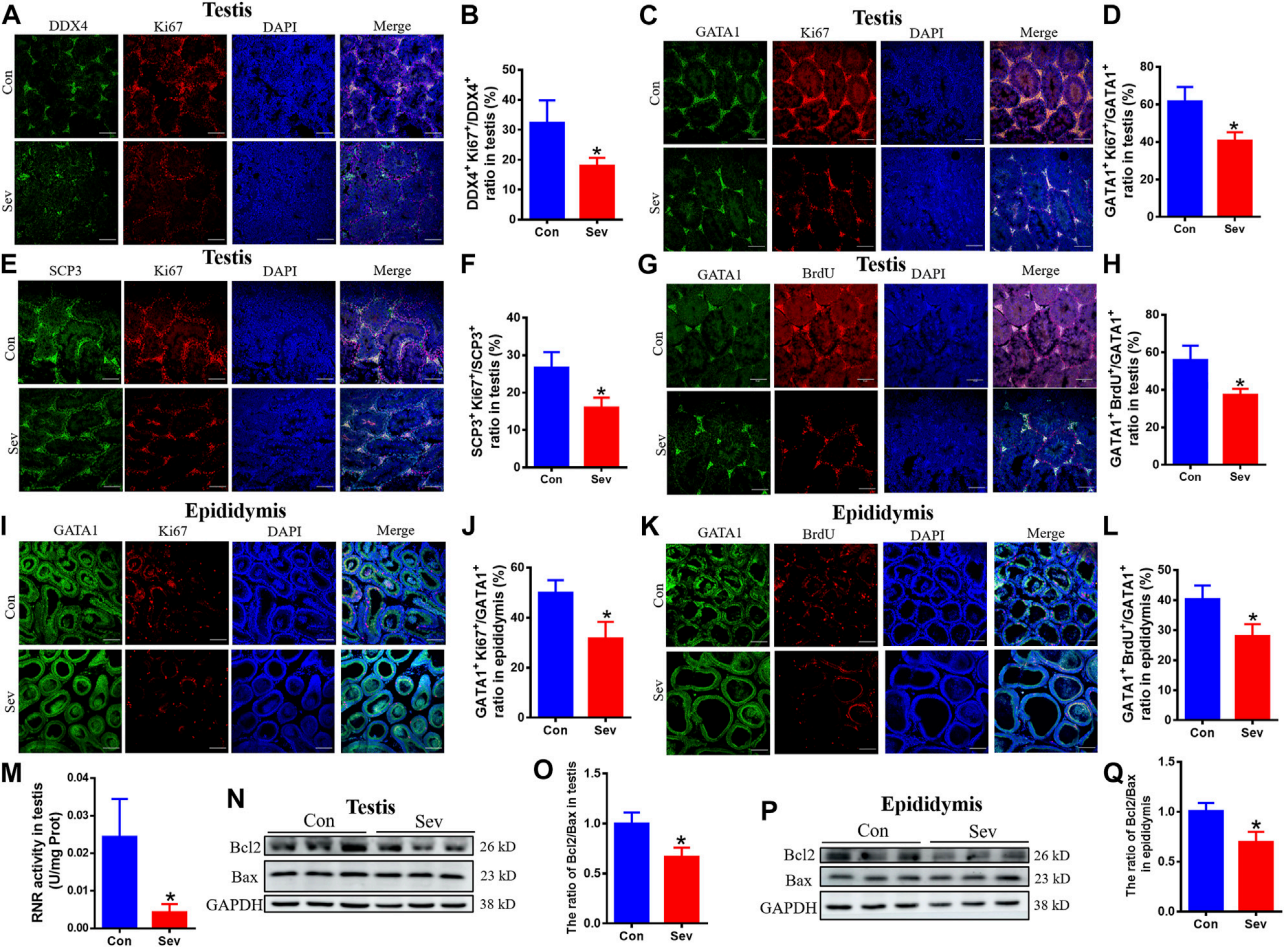
Effects of Sev on cell proliferation and apoptosis in testis and epididymis **(A)** and **(B)** The expression of Ki67 of sperm cells in testis was detected by immunofluorescence (n = 3, scale bar = 100 μm). **(C)** and **(D)** The expression of Ki67 of Sertoli cells in testis was detected by immunofluorescence (n = 3, scale bar = 100 μm). **(E)** and **(F)** The expression of Ki67 of primary spermatocytes in testis was detected by immunofluorescence (n = 3, scale bar = 100 μm). **(G)** and **(H)** The cell proliferation of Sertoli cells in testis (n = 3, scale bar = 100 μm). **(I)** and **(J)** The expression of Ki67 of Sertoli cells in epididymis was detected by immunofluorescence (n = 3, scale bar = 100 μm). **(K)** and **(L)**: The cell proliferation of Sertoli cells in epididymis (n = 3, scale bar = 100 μm). **(M)**: The effect of Sev on activity of RNR in testis (n = 5). **(N)** and **(O)**: Effects of Sev on the expression of Bcl2 and Bax in testis (n = 3). **(P)** and **(Q)**: Effects of Sev on the expression of Bcl2 and Bax in epididymis (n = 3). Data are expressed as mean ± SD. *p < 0.05 compare to that of control group.

Cell proliferation and apoptosis are the key factors that determine the number of cells. Ki67 was an antigen associated with proliferating cells, whose function was closely related to mitosis and is indispensable in cell proliferation. Thus, Ki67 was a biomarker representing cell proliferation. SCP3 and GATA1 were the markers of primary spermatocytes and Sertoli cells (LaVoie, 2003; Li et al., 2016), respectively. BrdU label could reflect the DNA synthesis status. To evaluate the effect of iron deficiency on cell proliferation of germ cells, primary spermatocyte and Sertoli cells in testis and epididymis, we tested the co-staining of DDX4^+^/Ki67^+^, SCP3^+^/Ki67^+^ GATA1^+^/Ki67^+^ and GATA1^+^/BrdU^+^. Our results revealed that the number of DDX4^+^, SCP3^+^ and GATA1^+^ significantly decreased, as shown in Figures 3A–L, indicating that iron deficiency inhibited spermatogenesis. RNR is the key enzyme in DNA replication, which is the basis of cell proliferation. Iron is an important cofactor of RNR. Thus, we assayed the activity of RNR in testis. Our results showed that Sev potentially decreased the RNR activity, as shown in Figure 3M. Further, we tested the expression of Bcl2 and Bax in testis and epididymis to evaluate the effect of iron deficiency on cellular apoptosis. We found that Sev significantly decreased the ratio of Bcl2/Bax in Figures 3N–Q. All these results indicated that iron deficiency not only inhibited sperm cell proliferation, but promoted the cellular apoptosis in testis and epididymis, which might be one of the reasons for the sperm count decrease.

### Sev decreased the respiration effect and ATP production

**FIGURE 4:**
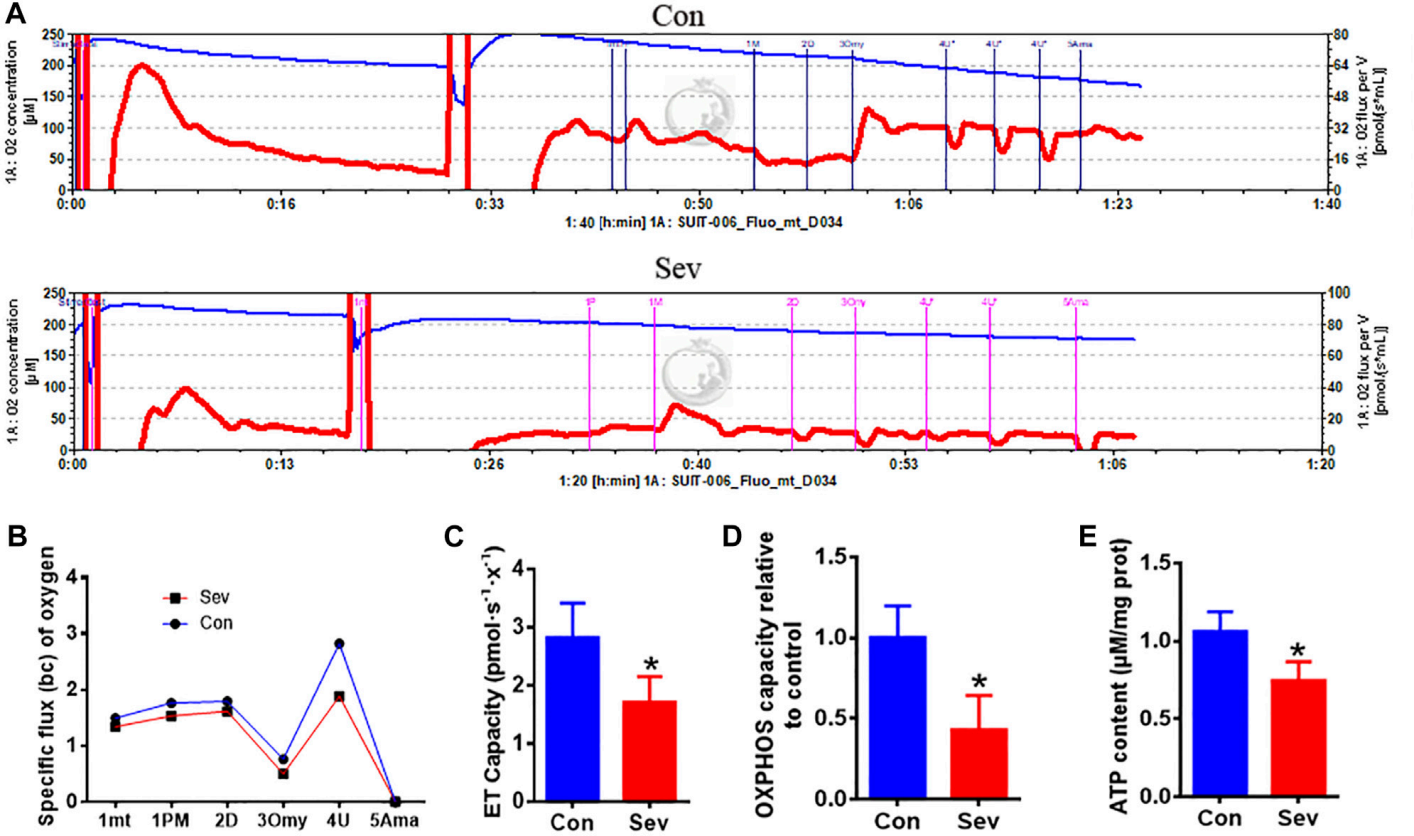
Sev reduced oxygen consumption, inhibited electron transport and oxidative phosphorylation (OXPHOS) levels, and decreased ATP production in testis. **(A)** Sev decreased oxygen consumption in testis. **(B)** Panel B was the quantitative statistical result of Figure **(A)**. **(C)** and **(D)** Sev inhibited electron transport along the respiratory chain (n = 5) and OXPHOS (n = 5) in testis. **(E)** Sev decreased ATP production in testis (n = 5). Data were expressed as mean ± SD. *p < 0.05 compare to that of control group. mt: mitochondrial; P M: pyruvate and malate; D: ADP; Omy: oligomycin; U: FCCP; Ama: antimycin A.

Considering iron’s effect on electric transfer in respiration, we hypothesized that iron deficiency influenced the respiration effect. Thus, we used O2k to measure the respiration effect under Sev treatment. Our results showed that Sev significantly decreased the specific oxygen flux as shown in Figures 4A,B. The decrease in oxygen consumption further inhibited the electron transport (ET) capacity (*p* < 0.05) and OXPHOS levels (*p* < 0.05) which suppressed the ATP production (*p* < 0.05) in epididymis as shown in Figures 4C–E. These data might explain the reason that caused decrease in the sperm mobility.

### Sev impaired the tight junctions of testis and epididymis

**FIGURE 5:**
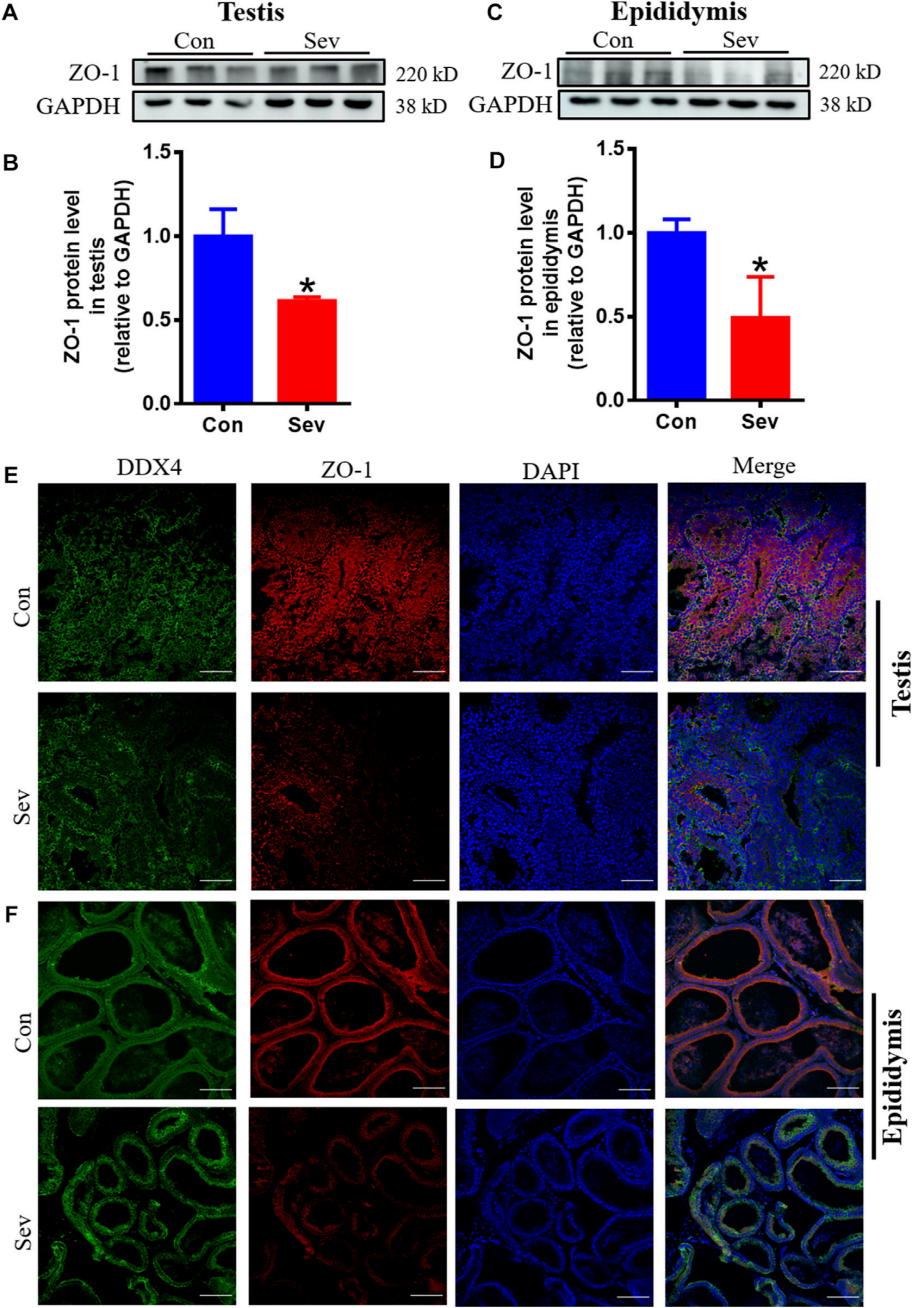
Sev impaired the tight junction of blood-testis barrier. **(A)** and **(B)** Sev inhibited ZO1 expression in testis by Western blot detection. **(C)** and **(D)** Effects of Sev on ZO1 expression in epididymis. **(E)** and **(F)** The co-expression of DDX4 and ZO-1 was detected by immunofluorescence in testis and epididymis. Data are expressed as mean ± SD. *p < 0.05 compare to that of control group.

The tight intercellular junctions in the blood-testosterone barrier played an important role in regulating the movement of substances into and out of the testis and ZO1 is the biomarker of tight junctions (Schwayer et al., 2019). In order to evaluate the effect of Sev on the junctions, we investigated the expression of ZO1. The results of both Western blot and immunofluorescence co-containing showed that Sev not only downregulated the expression of ZO1 in testis and epididymis, but decreased the expression in sperm cells as shown in Figures 5A–F, indicating that tight junctions impairment affected the iron influx and the function of testis and epididymis.

### Iron supplementation inhibited the damaging effects of Sev on testis and epididymis

**FIGURE 6:**
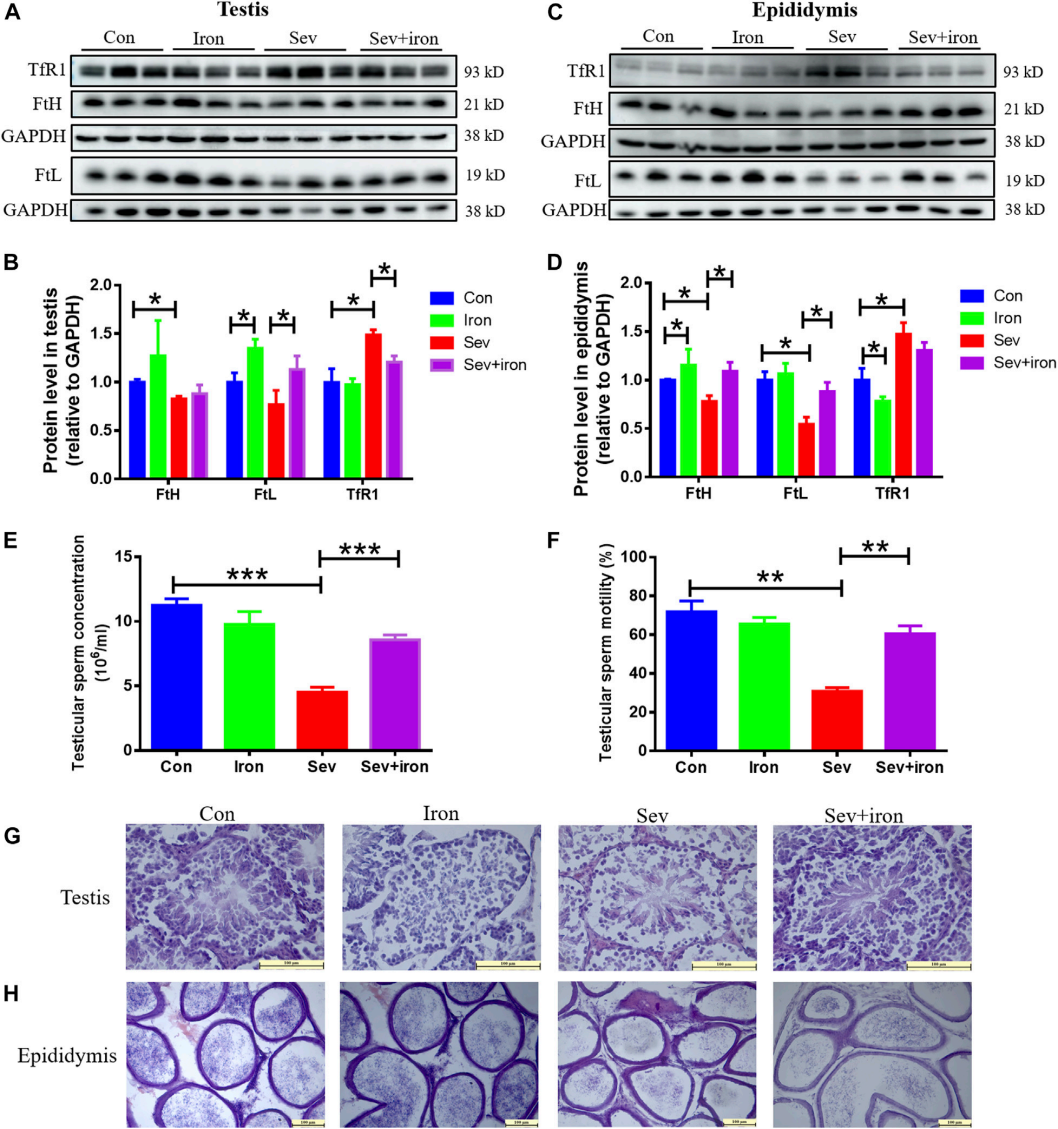
Iron therapy ameliorated iron metabolism disorder, the testicular and epididymal damage caused by Sev and improved sperm activity. **(A)** and **(C)**: Iron supplementation inhibited Sev-induced iron metabolism disorder in testis and epididymis. **(B)** and **(D)**: Panel B (n = 3) and **(D)** (n = 3) were the quantitative statistical results of Figure **(A)** and **(C)**. **(E)** and **(F)**: Iron supplementation increased the sperm concentration (n = 3) and improved the sperm motility Sev-induced (n = 3). **(G)** and **(H)**. Iron supplementation suppressed the Sev-induced structural damage of testis and epididymis (n = 3, 10X: Scale bar = 100 μm, 20X:Scale bar = 100 μm). Data were expressed as mean ± SD. *p < 0.05, **p < 0.01, ***p < 0.01.

Sev can cause damage to testis and epididymis through iron deficiency; we conducted an iron supplementation experiment to regulate iron homeostasis to study the effect of iron supplementation. Our results showed that iron supplementation inhibited the decrease in FtH and FtL, increase in TfR1 induced by Sev in testis and epididymis as shown in Figures 6A–D. Meanwhile, iron supplementation activated the sperm motility, increased the sperm count, and suppressed the impairment of Sev on testis and epididymis as shown in Figures 6E–H. These data showed that maintaining iron homeostasis can significantly inhibit Sev damage to male reproductive organs.

## Discussion

Some reports suggested that incidences of infertility, spontaneous abortus, congenital anomaly, germ cell injury, sperm cell DNA damage, and morphological changes in sperm cells are increased due to exposure to anesthetic gases such as Sev (Ceyhan et al., 2005; Kaymak et al., 2012). However, the exact mechanisms were not clear yet. Our previous studies demonstrated that Sev can induce central nervous system iron metabolism disorders and cause cognitive impairment in mice (Zuo et al., 2020; Ge et al., 2021). Thus, we hypothesized that Sev affected spermatogenesis and fertility by inducing iron metabolism disorders. Behavioral results showed that Sev did cause a short-term reduction in the number of mice sperm, sperm motility, and the number of offspring significantly decreased (Figure 1). These effects were due to damaged tissue in the testis and epididymis. In the testis, iron was concentrated in the seminiferous tubules by transferring and was an important element in controlling the interaction between Sertoli and germ cells in the testis (Intragumtornchai et al., 1988). Iron deficiency affected the levels of plasma luteinizing hormone and testosterone in the adult male rat (Intragumtornchai et al., 1988), indicating that iron was one of the key factors in spermatogenesis. Here, we demonstrated for the first time that Sev inhibited the spermatogenesis and development through inducing iron deficiency in testis and epididymis. Sev significantly decreased the gene expression of FtH and FtL in testis and epididymis. Meanwhile, Sev increased the expression of TfR1. The results suggested that Sev caused the iron deficiency in testis and epididymis which was further confirmed by ICP-MS and Perl’s staining. DDX4 was a marker of germ cells in testis and epididymis. In order to evaluate the effect of Sev on the iron levels of germ cells, we further tested the expression of FtH and FtL in germ cells of testis and epididymis. The results showed that Sev downregulated the expression of FtH and FtL in germ cells (Figure 2).

Previous studies reported that Ki67 was one of the markers in cell proliferation and iron was a cofactor of RNR (Sanvisens et al., 2011; Li et al., 2019). SCP3 and GATA1 were the markers of primary spermatocyte and Sertoli cells, respectively. Sertoli cells are the cells that provide protection and nutrition for the developing sperm. All stages of sperm development take place on the surface of Sertoli cells. We performed the immunofluorescence double staining to assay the number of sperm cells, Sertoli cells and primary spermatocytes and the DNA synthesis in testis and epididymis. We found that Sev significantly decreased the number of sperm cells, Sertoli cells, and primary spermatocytes by inhibiting DNA synthesis in testis and epdidymis (Figure 3).

RNR is a multi-subunit enzyme responsible for catalyzing the rate-limiting step in the synthesis of DNA (Chakraborty et al., 2022). We found that iron deficiency suppressed the activity of RNR in testis. The main structure of the testis is made up of spermatogenic tubules which contain spermatogenic cells, peritubular cells and Sertoli cells and account for two-thirds of the total volume of the testis (Neto et al., 2016; Nakata, 2019). In the adult testes, mature sertoli cells maintain spermatogonia and support spermatogenesis during the entire lifetime (Yokonishi and Capel, 2021). According to the above results, we speculated that Sev maybe inhibited spermatogenesis mainly through affecting Sertoli cell development. To explain the low sperm count, our results also showed that Sev induced cellular apoptosis through Bcl2/Bax pathway (Figure 3). Sperm motility is powered by ATP, and its motility is a key indicator of sperm development quality. In animal cells, ATP is mainly derived from oxidative phosphorylation on mitochondria. Electron transport along the respiratory chain is the main pathway of oxidative phosphorylation to produce ATP. Iron is an essential cofactor for the activity of ribonucleic acid reductase and plays a key role in electron transport in the mitochondrial respiratory chain. To evaluate the effect of iron deficiency on ATP production, we tested the flux of oxygen, electron transport (ET) capacity and oxidative phosphorylation (OXPHOS) capacity and ATP contents. Results revealed that Sev decreased the oxygen consumption, inhibited the ET capacity and OXPHOS levels which led to lower ATP levels (Figure 4). Sevoflurane-induced the iron deficiency and reduced ATP content may be one of the reasons of low sperm motility and cellular apoptosis. OXPHOS involves the function of the complex I-IV on the mitochondrial respiratory chain which contains many proteins or enzymes. In the future, we need to clarify exactly how Sev affects the function of the respiratory chain or the expression of which proteins in testis.

ZO1 is the key molecule in the tight junction of the blood-testis barrier and plays an important role in the regulation of substance exchange. Here, we showed that Sev downregulated the expression of ZO1in testis and epididymis or between sperm cells, indicating that Sev could impaired the blood–testis barrier, which further affected spermatogenesis and development (Figure 5). Interestingly, some of these damaging effects above could be ameliorated by iron supplementation (Figure 6).

## Conclusion

In conclusion, in the short term, male mice exposed to Sev can affect spermatogenesis and fertility through iron deficiency of epididymis and testis. Iron supplementation before anesthesia may effectively inhibit the formation and development of spermatozoa damaged by Sev. Furthermore, the damaging effects could be ameliorated by iron supplementation.

## Data Availability

The original contributions presented in the study are included in the article/Supplementary Material, further inquiries can be directed to the corresponding authors.

## Abbreviations

Sev, sevoflurane; TfR1, transferrin receptor 1; FtH, heavy chain ferritin; FtL, light chain ferritin; Brdu, bromodeoxyuridine; ET, electron transport; FpN1, Ferroportin1; RNR, ribonucleotide reductase; OXPHOS, oxidative phosphorylation; FpN1, Ferroportin1.
